# Cytochrome *b*_*5*_ impacts on cytochrome P450-mediated metabolism of benzo[*a*]pyrene and its DNA adduct formation: studies in hepatic cytochrome *b*_*5*_/P450 reductase null (HBRN) mice

**DOI:** 10.1007/s00204-018-2162-7

**Published:** 2018-01-24

**Authors:** Lindsay Reed, Iveta Mrizova, Frantisek Barta, Radek Indra, Michaela Moserova, Klaus Kopka, Heinz H. Schmeiser, C. Roland Wolf, Colin J. Henderson, Marie Stiborova, David H. Phillips, Volker M. Arlt

**Affiliations:** 1 Department of Analytical, Environmental and Forensic Sciences, MRC-PHE Centre for Environment and Health, King’s College London, Franklin-Wilkins Building, 150 Stamford Street, London, SE1 9NH UK; 2 Department of Biochemistry, Faculty of Science, Charles University, Albertov 2030, 128 40 Prague 2, Czech Republic; 3 Division of Radiopharmaceutical Chemistry, German Cancer Research Center (DKFZ), Im Neuenheimer Feld 280, 69120 Heidelberg, Germany; 4 Division of Cancer Research, Jacqui Wood Cancer Centre, School of Medicine, University of Dundee, Ninewells Hospital, Dundee, DD1 9SY UK

## Abstract

Benzo[*a*]pyrene (BaP) is an environmental pollutant that, based on evidence largely from in vitro studies, exerts its genotoxic effects after metabolic activation by cytochrome P450s. In the present study, Hepatic Reductase Null (HRN) and Hepatic Cytochrome *b*_*5*_/P450 Reductase Null (HBRN) mice have been used to study the role of P450s in the metabolic activation of BaP in vivo. In HRN mice, cytochrome P450 oxidoreductase (POR), the electron donor to P450, is deleted specifically in hepatocytes. In HBRN mice the microsomal haemoprotein cytochrome *b*_*5*_, which can also act as an electron donor from cytochrome *b*_*5*_ reductase to P450s, is also deleted in the liver. Wild-type (WT), HRN and HBRN mice were treated by i.p. injection with 125 mg/kg body weight BaP for 24 h. Hepatic microsomal fractions were isolated from BaP-treated and untreated mice. In vitro incubations carried out with BaP-pretreated microsomal fractions, BaP and DNA resulted in significantly higher BaP–DNA adduct formation with WT microsomal fractions compared to those from HRN or HBRN mice. Adduct formation (i.e. 10-(deoxyguanosin-*N*^2^-yl)-7,8,9-trihydroxy-7,8,9,10-tetrahydro-BaP [dG-*N*^2^-BPDE]) correlated with observed CYP1A activity and metabolite formation (i.e. BaP-7,8-dihydrodiol) when NADPH or NADH was used as enzymatic cofactors. BaP–DNA adduct levels (i.e. dG-*N*^2^-BPDE) in vivo were significantly higher (~ sevenfold) in liver of HRN mice than WT mice while no significant difference in adduct formation was observed in liver between HBRN and WT mice. Our results demonstrate that POR and cytochrome *b*_*5*_ both modulate P450-mediated activation of BaP in vitro. However, hepatic P450 enzymes in vivo appear to be more important for BaP detoxification than its activation.

**Electronic supplementary material:**

The online version of this article (10.1007/s00204-018-2162-7) contains supplementary material, which is available to authorized users.

## Introduction

Polycyclic aromatic hydrocarbons (PAHs) are ubiquitous in the environment as a result of their formation by the incomplete combustion of organic matter (Baird et al. 2005; IARC 2010). Benzo[*a*]pyrene (BaP) is the most commonly studied and measured of the PAHs (Arlt et al. 2015b; Krais et al. 2016; Labib et al. 2012, 2016; Siddens et al. 2012; Wohak et al. 2016; Long et al. 2017) and it is classified by the International Agency for Research on Cancer (IARC) as a human carcinogen (Group 1). The unavoidable exposure of the human race to PAHs makes a substantial contribution to the overall burden of cancer in humans (Baird et al. 2005; IARC 2010; Phillips and Venitt 2012). A major source of exposure to BaP is tobacco smoke (Alexandrov et al. 2016); however, for non-smokers the principal sources can be dietary (Phillips 1999) and from exposure to air pollution (IARC 2016).

**Fig. 1:**
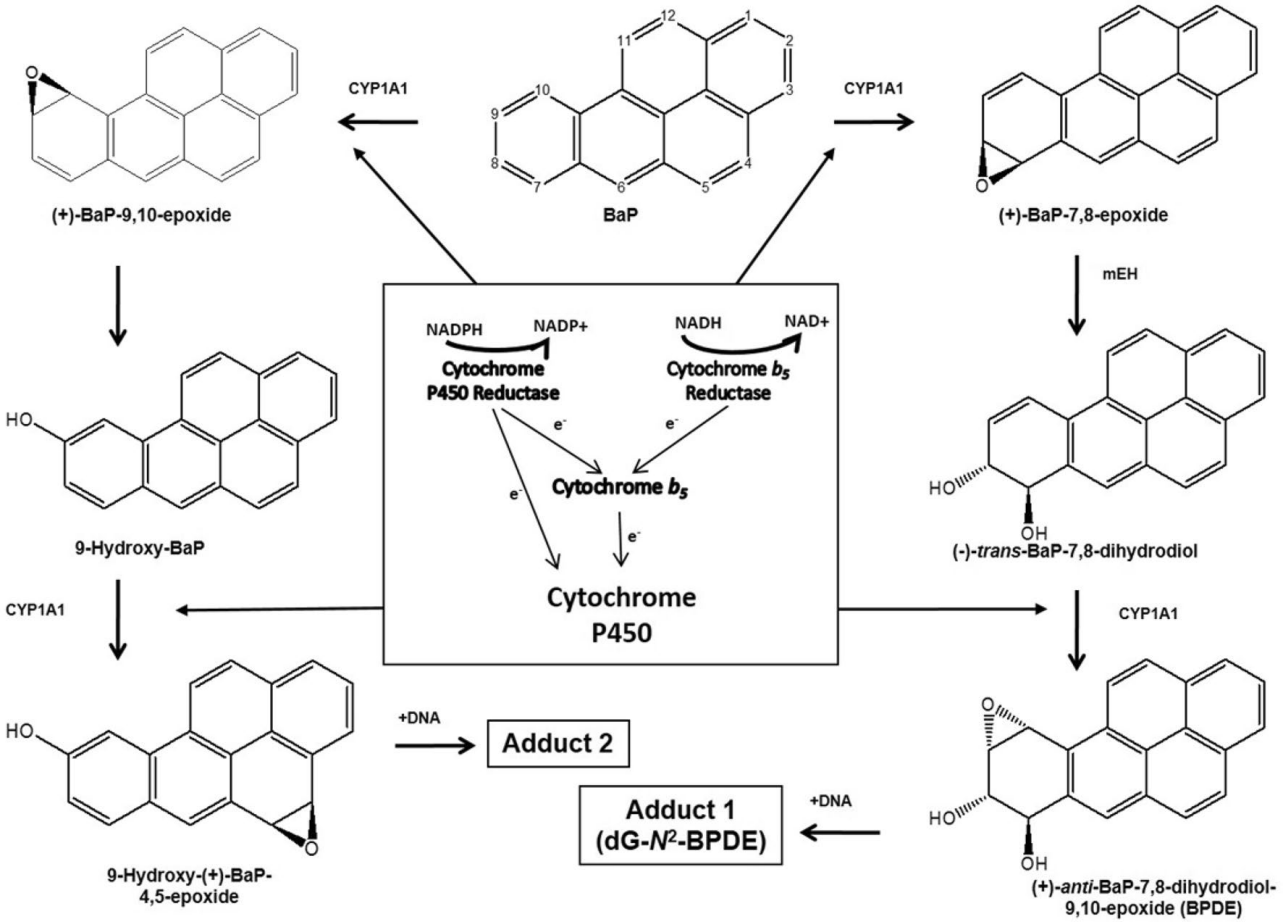
Pathways of BaP biotransformation and BaP–DNA adduct formation catalysed by CYP1A1 and mEH. The three-stage pathway, involving mEH, forming the ultimately reactive species BPDE that binds to guanine to form the dG-*N*^2^-BPDE adduct (adduct 1) is shown on the right. The two-stage pathway that does not involve mEH forms the second adduct (i.e. adduct 2) seen in in vitro studies is shown on the left; the structure of the adduct has not yet been elicited, the adduct is probably derived from the reaction of 9-hydroxy-BaP-4,5-epoxide with guanine residues in DNA. The diagram in the centre shows the roles of POR, Cyb5R and Cyb5 as electron donors to P450 enzymes such as CYP1A1 that are central to the biotransformation of BaP

BaP is a procarcinogen that requires metabolic activation before reacting with DNA and exerting its genotoxic effects (Luch and Baird 2005; Phillips 2005). It is activated via cytochrome P450 (P450)-dependent monooxygenases with CYP1A1 and CYP1B1 playing the major role. BaP is oxidised initially to BaP-7,8-epoxide, which is then converted to BaP-7,8-dihydrodiol by epoxide hydrolase (Baird et al. 2005; Luch and Baird 2005) (Fig. 1). BaP-7,8-dihydrodiol then undergoes further bioactivation by CYP1A1 and CYP1B1 to form the ultimately reactive species, BaP-7,8-dihydrodiol-9,10-epoxide (BPDE) (Fig. 1). BPDE reacts with DNA to form principally the pre-mutagenic adduct 10-(deoxyguanosin-*N*^2^-yl)-7,8,9-trihydroxy-7,8,9,10-tetrahydro-BaP (dG-*N*^2^-BPDE) (Arlt et al. 2008; Kucab et al. 2015; Long et al. 2016).

P450 enzymes play an important role in the bioactivation of many carcinogens (Nebert and Dalton 2006; Rendic and Guengerich 2012) and a number of transgenic mouse lines (e.g. *CYP*-knockout or CYP-humanised) have been developed to study the contribution of individual P450 enzymes to chemical-induced genotoxicity and carcinogenesis (Arlt et al. 2011a; Buters et al. 2002; Kimura et al. 1999, 2003; Stiborova et al. 2012; Uno et al. 2004). However, due to the large number of CYP isoforms and their overlapping substrate specificities, it has been difficult to determine the in vivo role of P450 enzymes as a whole. We have used the Hepatic P450 Reductase Null (HRN) mouse model as an approach to overcoming this limitation (for review see Arlt et al. 2015a). HRN mice have a deletion in their hepatocytes of P450 oxidoreductase (POR), the predominant electron donor to P450 enzymes, specifically (Henderson et al. 2003). Experiments with HRN mice have indicated that hepatic P450 enzymes play a more important role in the detoxification of BaP in vivo despite their importance for BaP activation in vitro (Arlt et al. 2008), because HRN mice treated with BaP formed up to 13-fold higher levels of BaP–DNA adducts than wild-type (WT) mice. Further investigations involving cellular localisation of BaP–DNA adducts by immunohistochemistry showed that HRN mice have ample capacity for the formation of BaP–DNA adducts (i.e. dG-*N*^2^-BPDE) in liver; no differences in BaP–DNA adduct formation was observed between hepatocytes (i.e. POR-deficient cells) and non-hepatocytes (i.e. POR-proficient cells) (Arlt et al. 2012). This paradoxical result is echoed by other studies with *Cyp1a1(-*/*-)* knockout mice (Uno et al. 2004), which also indicate the role of CYP1A1 in BaP detoxification in vivo (Nebert et al. 2013).

Although the specific enzyme(s) involved in the generation of BaP–DNA-binding species in the liver of HRN mice is not known, it is clear that the process does not produce a different reactive species from that formed in WT mice (Arlt et al. 2008, 2012). One hypothesis is that another electron donor to P450 enzymes may contribute to BaP–DNA adduct formation in the livers of HRN mice. Although POR is viewed as the predominant electron donor to P450 enzymes (Guengerich 2008), cytochrome *b*_*5*_ (Cyb5) can also act as the electron donor both in vitro and in vivo (Finn et al. 2008; Yamazaki et al. 2002). Cyb5 can modulate P450 activity in three ways: (1) by direct transfer of both electrons via cytochrome *b*_*5*_ reductase (Cyb5R) in a pathway independent of POR (Yamazaki et al. 1996a, b); (2) by the transfer of the second and rate-limiting electron from either POR or Cyb5R (Hildebrandt and Estabrook 1971); or (3) by acting as an allosteric modifier of the enzyme in a non-catalytic role that can enhance reactions for many, but not all, P450 enzymes (Yamazaki et al. 2002). Cyb5 is both substrate and enzyme specific, and has been shown to both stimulate and inhibit P450 reactions. Therefore, it is difficult to predict the contribution of Cyb5 to xenobiotic metabolism.

Previously we found higher protein expression of Cyb5 in HRN mice than in WT mice after repeated BaP exposure (Arlt et al. 2012). Similar results were found for microsomal epoxide hydrolase (mEH), another important enzyme involved in the formation of the DNA-reactive intermediate BPDE (Arlt et al. 2012). To investigate the role of Cyb5 in the metabolic activation of BaP in vitro, studies using reconstituted systems were carried out. The results showed that even under conditions where POR levels were low, CYP1A1, Cyb5, and mEH were able to activate BaP into reactive species that bind to DNA (Stiborova et al. 2014). Further, both NADPH (cofactor for the POR system) and NADH (cofactor for the Cyb5/Cyb5R system) stimulated CYP1A1-mediated BaP bioactivation in vitro, suggesting that the NADH/Cyb5R/Cyb5 system can act as sole electron donor to CYP1A1-catalysed BaP bioactivation (Stiborova et al. 2016a, b). All these findings indicate that even low POR expression in the livers of HRN mice (probably in non-parenchymal cells), in combination with the induction of Cyp1a1, Cyb5 and mEH by BaP, might be sufficient for efficient BaP bioactivation in vivo, replacing NADPH-dependent POR in the CYP1A1-catalysed activation of BaP (Stiborova et al. 2014). Given the in vitro data and the increased protein expression of Cyb5 in HRN mice after repeated BaP exposure (Arlt et al. 2012), it is possible that Cyb5 compensates for the absence of POR in the HRN mice and stimulates P450-mediated BaP bioactivation. By crossing HRN mice with the hepatic cytochrome *b*_*5*_ null (HBN) mouse, the hepatic cytochrome *b*_*5*_/P450 reductase null (HBRN) mouse model was developed (Henderson et al. 2013); these mice lack both POR and Cyb5 in the liver and have reduced hepatic P450 activity relative to the HRN mouse (Henderson et al. 2013). In the present study we have used both the HRN and HBRN mouse models to investigate the contribution of Cyb5 to the metabolic activation of BaP in vivo.

## Materials and methods

### Chemicals

Benzo[*a*]pyrene (BaP; CAS no. 50-32-8; purity > 96%) was purchased from Sigma-Aldrich (St Louis, MO). All other chemicals were of analytical purity or better.

### Animal treatment

All animal experiments were carried at the University of Dundee out under licence in accordance with the Animal (Scientifc Procedures) Act (1986), as amended by EU Directive 2010/63/EU, and with local ethical approval. HRN (*Por*^*lox*/*lox*^/*Cre*^*CYP1A1*^*)* mice and HBRN (*Cytb*_*5*_^*lox*/*lox*^/*Por*^*lox*/*lox*^±*Cre*^*ALB*^) mice on a C57BL/6 background were derived as described previously (Henderson et al. 2003, 2013). Animals were maintained in open-top cages, with free access to food (RM1 diet, Special Diet Services, Essex, UK) and water, and a 12-h light/dark cycle. Mice homozygous for the floxed *Por* locus (*Por*^*lox*/*lox*^) were used as WT. Groups of female HRN, HBRN and WT mice (3 months old, 25–30 g) were treated intraperitoneally (i.p.) with 125 mg/kg body weight (bw) (*n* = 3) of BaP for 1 day following a treatment protocol used previously to study BaP metabolism in HRN mice (Arlt et al. 2008, 2012). BaP was dissolved in corn oil at a concentration of 12.5 mg/ml. Control mice (*n* = 3) received solvent (corn oil) only. Animals were killed 24 h after the single dose and their tissues (liver, lung, forestomach, glandular stomach, kidney, spleen, small intestine, bladder and colon) were collected, snap-frozen and stored at − 80 °C until analysis.

### BaP–DNA adduct detection by ^32^P-postlabelling analysis

Genomic DNA from whole tissue was isolated by a standard phenol–chloroform extraction method and DNA adducts were measured for each DNA sample using the nuclease P1 enrichment version of the thin-layer chromatography (TLC)–^32^P-postlabelling method as described previously (Arlt et al. 2008, 2012). After chromatography, TLC plates were scanned using a Packard Instant Imager (Dowers Grove, IL, USA). DNA adduct levels were calculated as described (Phillips and Arlt 2014). Results were expressed as DNA adducts/10^8^ nucleotides.

### Preparation of microsomes

Hepatic microsomes from untreated or BaP-treated mice were isolated as described previously (Arlt et al. 2008). Microsomes were isolated from 3 pooled livers of each mouse model. Protein concentration in the microsomal fraction was measured using the bicinchoninic acid protein assay with bovine serum albumin as standard. Pooled microsomal fractions were used for further experiments.

### Enzyme activity assays and immunoblotting

The hepatic microsomal fractions were characterised for Cyp1a1 enzyme activity using Sudan I oxidation (Stiborova et al. 2002) and for Cyp1a1 and Cyp1a2 enzyme activities, we used 7-ethoxyresorufin *O*-deethylation (EROD) (Stiborova et al. 2005). POR enzyme activity was determined using cytochrome *c* (Arlt et al. 2003). Western blotting analysis using 4–12% bis–tris gradient gels and sodium dodecyl sulphate-polyacrylamide gel electrophoresis (SDS-PAGE) were carried out as described previously (Kucab et al. 2012). After migration, the proteins were transferred onto polyvinylidene difluoride (PVDF) membranes and the following primary antibodies were used: anti-Cyp1a1 1:1000 (sc-20772 (H-70), Santa Cruz Biotech), anti-POR (ab39995, Abcam), anti-Cyb5 1:750 (ab69801, Abcam), and anti-Cyb5R 1:1000 (ABIN453978, antibodies-online.com). The antibody to detect glyceraldehyde phosphate dehydrogenase (Gapdh) 1:25,000 (MAB374, Chemicon) was used as loading control. The secondary horseradish peroxidase-linked antibodies were as follows: anti-goat 1:10,000 (sc-2020, Santa Cruz) anti-rabbit 1:10,000 (#170–5046, BioRad). The antigen–antibody complex was visualised using SuperSignal^®^ West Pico Chemiluminescent Substrate Kit (Thermo Scientific).

### Microsomal incubations for BaP–DNA adduct formation

Incubation mixtures consisted of 50 mM potassium phosphate buffer (pH 7.4). Reduced nicotinamide adenine dinucleotide (NADPH) or reduced form of nicotinamide adenine dinucleotide (NADH) (1 mM in each case), pooled hepatic microsomal fraction (0.5 mg/ml protein) from HRN, HBRN and WT mice (either untreated or pretreated with BaP), 0.1 mM BaP (dissolved in 7.5 µl DMSO) and calf thymus DNA (0.5 mg) in a final volume of 750 µl. Incubations were carried out at 37 °C for 90 min (Arlt et al. 2008). Control incubations were carried out (1) without microsomes; (2) without NADPH or NADH; (3) without DNA and (4) without BaP. After incubation, DNA was isolated by a standard phenol–chloroform extraction method. BaP–DNA adduct formation was determined by ^32^P-postlabelling as described above.

### Microsomal incubations for studying BaP metabolism

Incubation mixtures contained 100 mM potassium phosphate buffer (pH 7.4), NADPH or NADH (1 mM), pooled hepatic microsomal fraction (0.5 mg/ml protein) and 50 µM BaP (dissolved in 5 µl DMSO) in a final volume of 500 µl. Incubations were carried out at 37 °C for 20 min. Control incubations were carried out (1) without microsomes; (2) without NADPH or NADH; (3) without BaP. After incubation, 5 µl of 1 mM phenacetin (PA) in methanol was added as an internal standard. BaP metabolites were extracted twice with ethyl acetate (1 ml), solvent evaporated to dryness, residues dissolved in 25 µl methanol and BaP metabolites were separated by high-performance liquid chromatography (HPLC) (Stiborova et al. 2014).

## Results

### Protein expression of XMEs

**Fig. 2:**
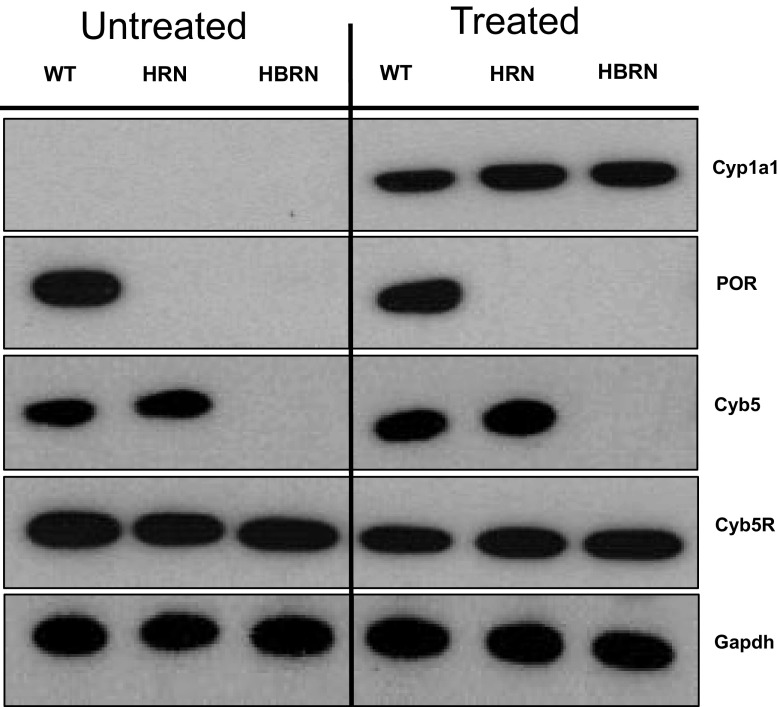
Western blot analysis of Cyp1a1, POR, Cyb5 and Cyb5R in the pooled microsomal fractions of untreated (lanes 1–3) and BaP-treated (lanes 4–6) WT, HRN and HBRN mice. Gapdh protein expression was used as a loading control. Representative images of the Western blotting are shown, and at least duplicate analysis was performed in separate experiments

Expression of electron donor proteins (i.e. POR, Cytb5, Cytb5R) associated with the mixed-function oxidase system (i.e. P450) was probed for in the hepatic microsomal fractions from BaP-treated and untreated (control) WT, HRN and HBRN mice (Fig. 2). POR was expressed only in WT mice and Cytb5 was expressed only in WT and HRN mice, as expected (Henderson et al. 2013, 2006). Cytb5R was expressed uniformly in all mouse lines. Treatment with BaP did not alter the levels of POR, Cytb5 and Cytb5R expression relative to controls. Cyp1a1 protein was greatly induced in all mouse lines by BaP treatment. The extent of Cyp1a1 protein induction by BaP was similar in all mouse lines. A previous study showed increased protein expression of Cyb5 in HRN mice after repeated (i.e. 5 days) BaP treatment (Arlt et al. 2012). No induction of Cyb5 was observed in HRN mice after BaP treatment here which is probably due to the single 24-h administration selected for this study.

### Enzyme activity of XMEs

**Fig. 3:**
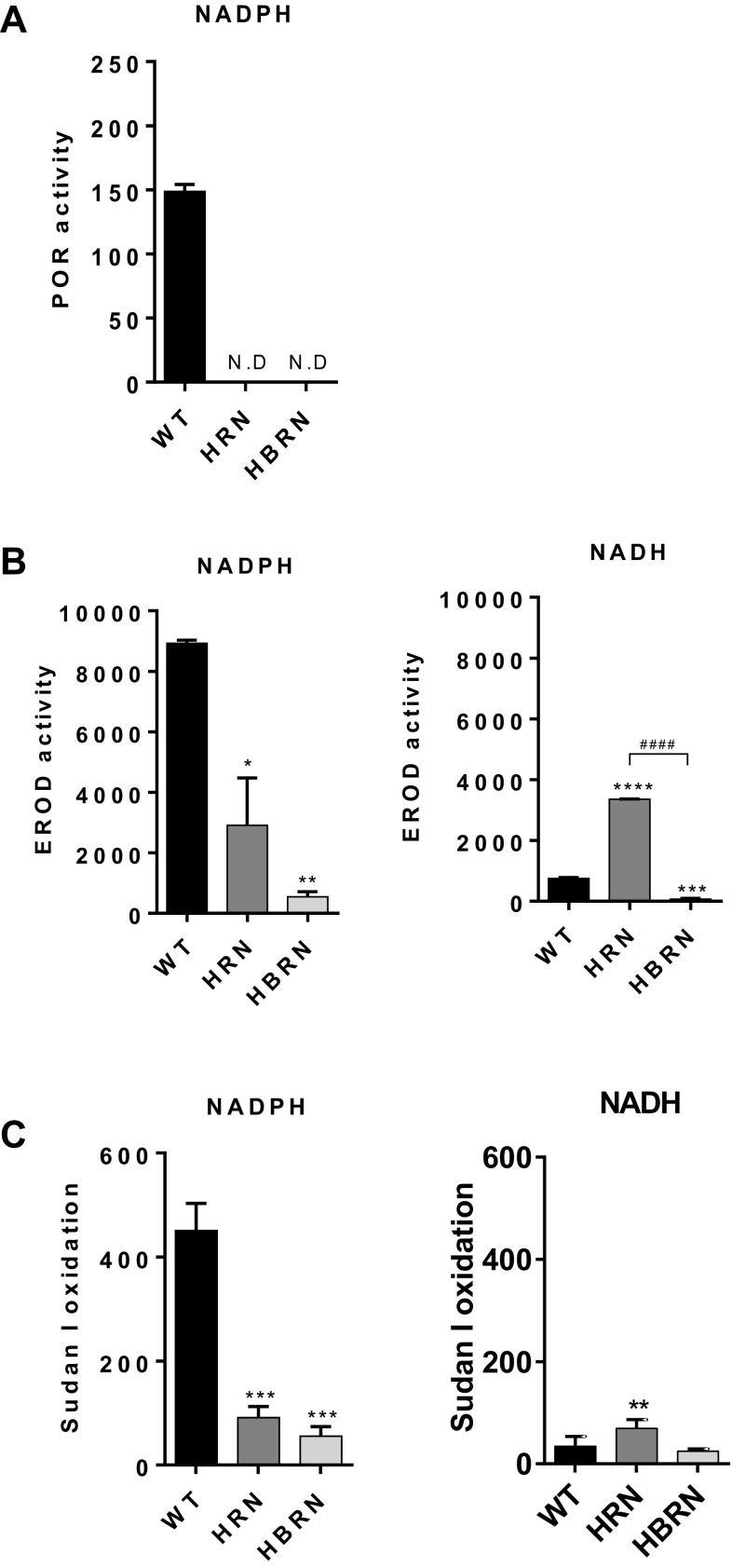
Enzyme activity in the pooled hepatic fractions of BaP-treated WT, HRN and HBRN mice using either NADPH or NADH as the cofactor. **a** POR activity was measured as nmol of cytochrome *c*/mg/min and was detected only in microsomal fractions from WT mice. **b** Cyp1a enzyme activity was determined using the EROD assay with activity being expressed as pmol of resorufin/mg protein/min. **c** Cyp1a1 enzyme activity was determined by the oxidation of Sudan I to hydroxylated metabolites with activity being measured as nmol of total C-hydroxylated metabolites/mg protein/min. Values are given as mean ± SD (*n* = 3). *ND* not detected. Statistical analysis was performed by one-way ANOVA with Tukey’s multiple comparison test (*, compared to WT; #, compared to HRN. *P ≤ 0.05 **P ≤ 0.01, ***P ≤ 0.001, ****P ≤ 0.0001)

POR activity was detected in the hepatic microsomal fractions from WT mice but not in knockout animals (Fig. 3a), as expected (Henderson et al. 2013, 2006). POR activity in BaP-treated WT mice was slightly lower than in untreated animals (compare Fig. 3a and Supplementary Fig. 1A).

We used EROD (Fig. 3b, Supplementary Fig. 1B) to measure Cyp1a1 and Cyp1a2 enzyme activity and Sudan I oxidation as a measure of Cyp1a1 enzyme activity (Fig. 3c, Supplementary Fig. 1C). Cyp1a activity was significantly higher in hepatic microsomes from mice treated with BaP than those from untreated mice, regardless of the enzyme cofactor (NADPH or NADH) used in the reaction mixture. When NADPH was used, hepatic microsomes from BaP-treated WT mice exhibited the highest levels of Cyp1a activity (Fig. 3b, c). Cyp1a activity was significantly lower in the hepatic microsomes from BaP-treated HRN mice which correlated with the lack of POR activity in these mice. Cyp1a activity in hepatic microsomes from BaP-treated HBRN mice was significantly lower compared to BaP-treated WT and HRN mice and showed the lowest level of Cyp1a activity when NADPH was present. For untreated mice Cyp1a activity was also lower in HRN and HBRN mice than in WT mice, but the effect was less pronounced compared with mice treated with BaP (Supplementary Fig. 1B and C).

When NADH was used in the reaction mixture, hepatic microsomes from BaP-treated HRN mice showed significantly higher Cyp1a activity than the hepatic microsomal fractions from BaP-treated WT and HBRN mice (Fig. 3b, c). Using EROD as a measure for Cyp1a activity, hepatic microsomes from BaP-treated HBRN mice exhibited the lowest level of Cyp1a activity in the presence of NADH (Fig. 3b). When Sudan I oxidation was used as a measure for Cyp1a1 activity, Cyp1a1 activity was similar in BaP-treated WT and HBRN mice (Fig. 3c). In untreated mice Cyp1a activity was lower in the absence of the electron donors when NADPH was used in the reaction mixture, with activity highest in WT mice and lowest in HBRN mice. When NADH was used in the reaction mixtures Cyp1a activity was highest in the HRN mice. Cyp1a activity measured by EROD activity was lowest in WT mice, but Cyp1a1 activity measured by Sudan I oxidation was lowest in the HBRN mice (Supplementary Fig. 1B and C).

### Analysis of BaP metabolites by HPLC

**Fig. 4:**
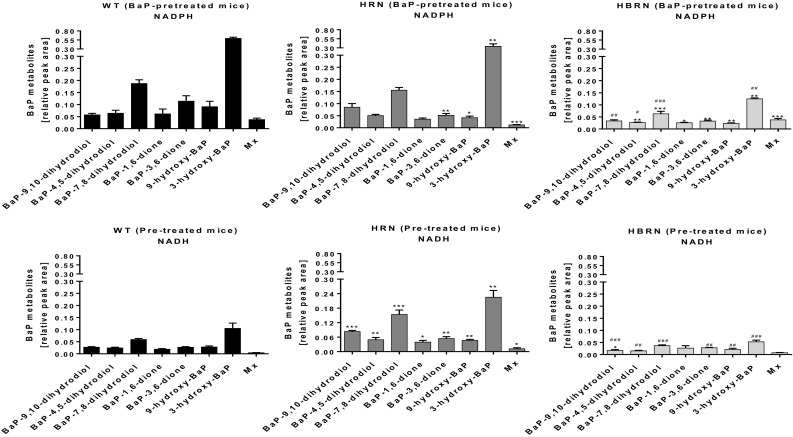
Detection of BaP metabolites on HPLC from in vitro incubations with BaP and hepatic microsomal fractions from BaP-pretreated WT, HRN and HBRN mice using either NADPH or NADH as cofactor. Values are given as mean ± SD (*n* = *3*). Statistical analysis was performed by one-way ANOVA with Tukey’s multiple comparison test (*, compared to WT; #, compared to HRN. *P ≤ 0.05, **P ≤ 0.01, ***P ≤ 0.001)

Hepatic microsomes isolated from WT, HRN and HBRN mice were incubated with BaP and analysed by HPLC to determine the BaP metabolite profile. The total formation of metabolites in vitro was the highest in the hepatic microsomal fraction from BaP-pretreated WT mice when NADPH was used as cofactor, around threefold higher than when NADH was used (Supplementary Fig. 2A). It is noteworthy that residual BaP and/or BaP metabolites were not detected in hepatic microsomes isolated from BaP-treated mice (Supplementary Fig. 4C). Representative HPLC chromatograms showing the BaP metabolite profiles are shown in Supplementary Fig. 4. Hydroxylated BaP metabolites and BaP-dihydrodiols, as well as BaP-diones, were identified and the structures are shown in Supplementary Fig. 5. We also detected a metabolite Mx whose structure has yet to be elucidated. The two major metabolites formed in all incubations were BaP-7,8-dihydrodiol and 3-hydroxy-BaP (Fig. 4). No BaP metabolites were detected by HPLC in control incubations without microsomes, without NADPH/NADH-generating system or without BaP (Supplementary Fig. 4C and D).

With NADPH as cofactor the overall formation of BaP metabolites was significantly lower in hepatic microsomal fractions from BaP-pretreated HRN mice with formation of BaP-3,6-dione, 9-hydroxy-BaP, 3-hydroxy-BaP and the unidentified metabolite Mx being significantly lower than with BaP-pretreated WT mice (Fig. 4). The lowest extent of overall BaP metabolite formation was in the hepatic microsomal fractions from BaP-pretreated HBRN mice (Supplementary Fig. 2A). All metabolites except for BaP-9,10-dihydrodiol were detected at significantly lower amounts compared with BaP-pretreated WT hepatic microsomes. BaP-9,10-dihydrodiol, BaP-4,5-dihydrodiol, BaP-7,8-dihydrodiol and 3-hydroxy-BaP were at lower levels than with BaP-pretreated HRN hepatic microsomes (Fig. 4).

When NADH was used as cofactor the overall formation of BaP metabolites in hepatic microsomal fractions from BaP-pretreated HRN mice was significantly higher than with fractions from BaP-pretreated WT mice with all metabolites being formed in higher quantities. There was no significant difference between overall metabolite formation in hepatic fractions from BaP-pretreated WT and HBRN mice (Supplementary Fig. 2A) with only BaP-9,10-dihydrodiol being significantly lower (Fig. 4). All metabolites except for BaP-1,6-dione and Mx were significantly lower compared with BaP-pretreated HRN hepatic microsomes (Fig. 4). The rate of BaP metabolism in the hepatic microsomal fractions correlated with the levels of Cyp1a enzymatic activity and the cofactor used (NADPH or NADH).

The overall formation of BaP metabolites in the hepatic microsomal fractions from untreated mice was highest in WT mice when NADPH was used in the reaction mixture, whereas when NADH was used the highest formation of metabolites was seen in HRN mice (compare Supplementary Fig. 2A and Fig. 3a). This pattern is similar to that observed in microsomes from BaP-pretreated mice, correlating with Cyp1a activity. However, the amount of metabolites detected was lower (Supplementary Fig. 6B).

### BaP–DNA adduct formation in vitro

We investigated the ability of hepatic microsomes isolated from control and BaP-treated WT, HRN and HBRN mice to catalyse BaP–DNA adduct formation in vitro (Supplementary Fig. 2B and 3B). The BaP–DNA adduct pattern obtained by ^32^P-postlabelling analysis from in vitro microsomal incubations consisted of up to two major spots (Supplementary Fig. 7). One spot (assigned adduct 1) was previously identified as 10-(deoxyguanosin-*N*^2^-yl)-7,8,9-trihydroxy-7,8,9,10-tetrahydrobenzo[*a*]pyrene (dG-*N*^*2*^-BPDE) (Arlt et al. 2008). The other spot (assigned adduct 2), which has not yet been fully structurally identified, is likely derived from the reaction of 9-hydroxy-BaP-4,5-epoxide with guanine residues in DNA (see Fig. 1) (Schoket et al. 1989; Nesnow et al. 1993; Fang et al. 2001). These two adducts result from the biotransformation pathways illustrated in Fig. 1. Essentially no BaP–DNA adducts were formed in control incubations with microsomes of BaP-pretreated WT mice without BaP, but were detectable in control incubations with microsomes of BaP-pretreated HRN and HRBN mice (Supplementary Fig. 7). These findings indicate that some BaP that was not detectable by HPLC is retained in hepatic microsomes of BaP-pretreated HRN and HRBN mice, being activated to form BaP–DNA adducts underlining the high sensitivity of the ^32^P-postlabelling method.

During in vitro incubations with WT microsomal fractions from BaP-pretreated mice and with NADPH as cofactor, adduct 2 was the predominant DNA adduct formed with the formation of dG-*N*^*2*^-BPDE (adduct 1) being much lower (Supplementary Fig. 8A). This was not seen with the hepatic microsomal fractions from BaP-pretreated HRN and HBRN mice, where formation of the dG-*N*^*2*^-BPDE adduct was greater than adduct 2 when NADPH was used in the reaction mix, and adduct 2 was not detected with NADH as cofactor (Supplementary Fig. 8B). When NADPH was used the greatest BaP–DNA adduct formation was seen with microsomal fractions from BaP-pretreated WT mice. There was a significant reduction in total DNA adduct formation in the microsomal fractions from BaP-pretreated HRN mice with a further, significant, reduction in adduct formation in microsomal incubation from BaP-pretreated HBRN mice (Supplementary Fig. 2B). When NADH was used, the highest total adduct formation was from the microsomal fractions from BaP-pretreated HRN mice with adduct formation from BaP-pretreated WT and HBRN microsomal fractions being significantly lower (Supplementary Fig. 2B). The total amount of BaP–DNA adduct formation in the hepatic microsomal fractions correlated with the amounts of BaP metabolites formed and the particular enzymatic cofactor used (see Supplementary Fig. 2A).

**Fig. 5:**
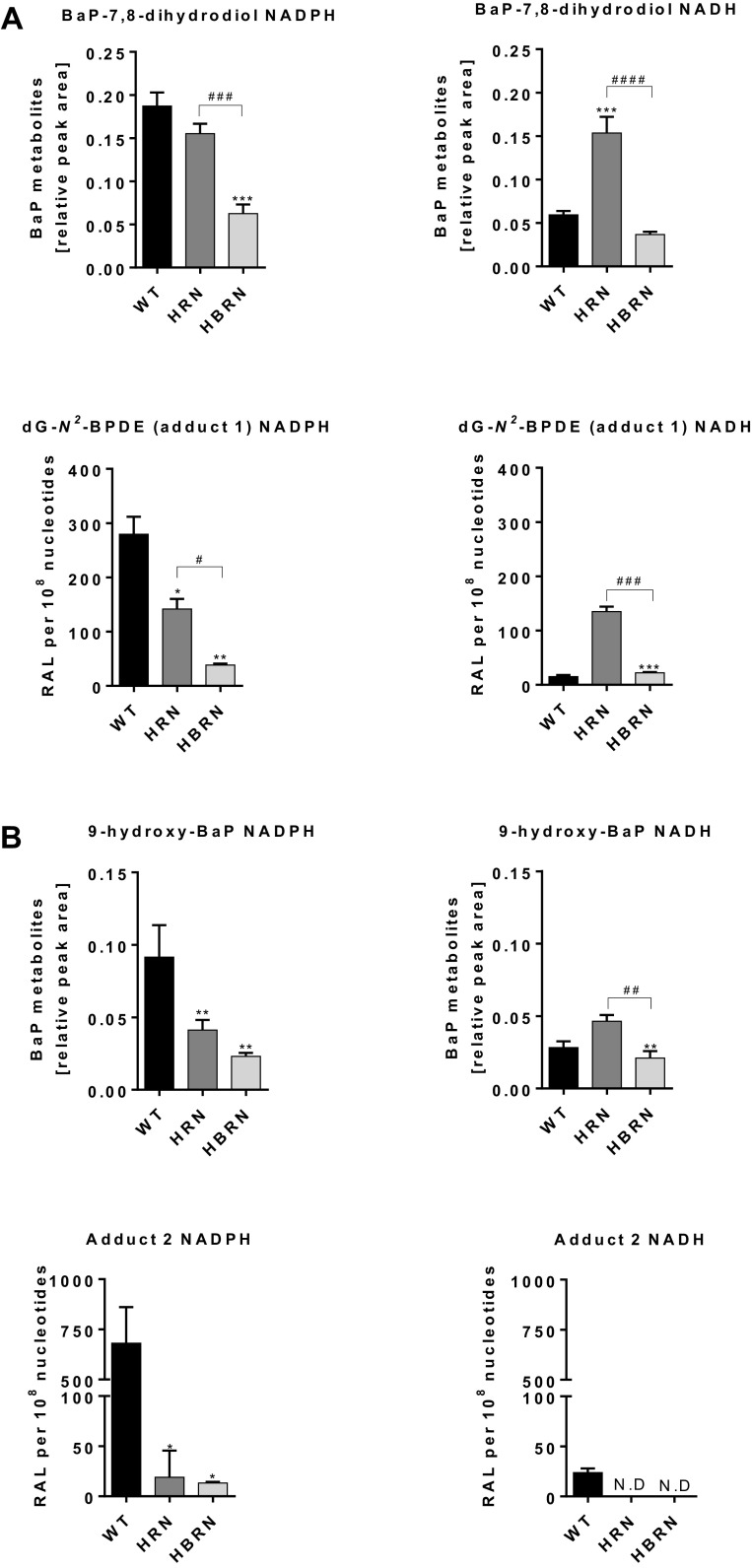
**a** Formation of BaP-7,8-dihydrodiol and dG-*N*^2^-BPDE (adduct 1) during in vitro incubations with hepatic microsomal fractions from BaP-pretreated WT, HRN and HBRN mice using either NADPH or NADH as cofactor. **b** Formation of 9-hydroxy-BaP and adduct 2 during in vitro incubations with hepatic microsomal fractions from BaP-pretreated WT, HRN and HBRN mice using either NADPH or NADH as cofactor. Values are given as mean ± SD (*n* = 3). *ND* not detected. Statistical analysis was performed by one-way ANOVA with Tukey’s multiple comparison test (*, compared to WT; #, compared to HRN. *P ≤ 0.05, **P ≤ 0.01, ***P ≤ 0.001, ****P ≤ 0.0001)

Levels of adduct 1 (dG-*N*^2^-BPDE) in vitro strongly correlated with the amounts of BaP-7,8-dihydrodiol formed in hepatic microsomal fractions from BaP-pretreated mice, both with NADPH and NADH as cofactor (Fig. 5a). When NADPH was used as cofactor, relative to WT, the formation of BaP-7,8-dihydrodiol was lower in HRN microsomal fractions and lowest in HBRN microsomal fractions, correlating with dG-*N*^2^-BPDE (adduct 1) formation in these microsomal fractions. The highest amounts of BaP-7,8-dihydrodiol were observed in HRN microsomal fractions with NADH as cofactor, with similar amounts being formed in microsomal fractions from WT and HBRN mice (Fig. 5a).

Using NADPH as cofactor the amounts of 9-hydroxy-BaP correlated with the levels of adduct 2 in hepatic microsomal fractions from BaP-pretreated mice (Fig. 5b). The amounts of 9-hydroxy-BaP were also the highest in HRN microsomal fractions when NADH was used as cofactor (Fig. 5b). As adduct 2 was not detectable in microsomal fractions of either HRN or HBRN BaP-pretreated mice no correlation to the amounts of 9-hydroxy-BaP formed was found. Because DNA adduct formation was quite low in microsomal fractions from untreated mice (Supplementary Fig. 8), no correlation to metabolite formation was attempted.

### BaP–DNA adduct formation in vivo

**Fig. 6:**
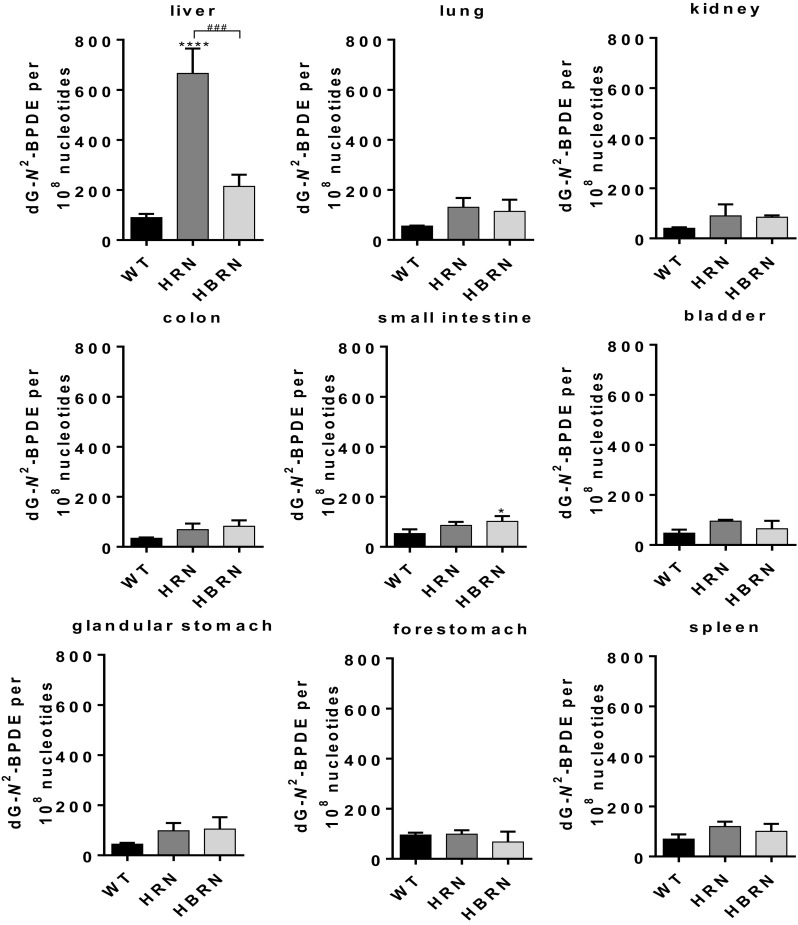
Quantitative TLC ^32^P-postlabelling analysis of dG-*N*^2^-BPDE adducts in individual organs of WT, HRN and HBRN mice treated i.p. with 125 mg/kg bw BaP for 24 h. Values are given as mean ± SD (*n* = 3). Statistical analysis was performed by one-way ANOVA with Tukey’s multiple comparison test (*, compared to WT; #, compared to HRN. *P ≤ 0.05, ***P ≤ 0.001, ****P ≤ 0.0001)

The BaP–DNA adduct pattern obtained from in vivo treatments consisted of one major adduct spot, dG-*N*^*2*^-BPDE (Supplementary Fig. 9). No DNA adducts were detected in tissues of untreated mice (data not shown). Of the organs tested (liver, lung, kidney, small intestine, spleen, forestomach, glandular stomach, colon and bladder) there was no significant difference exhibited between the mouse models with the exception of the small intestine and the liver (Fig. 6). In the small intestines there were significantly higher levels of BaP–DNA adducts in HBRN mice than in WT mice. BaP–DNA adduct formation in the livers of HRN mice was ~ sevenfold higher than in WT mice. BaP–DNA adduct formation in HBRN mice was significantly lower than that observed in HRN mice, although there is no significant difference between WT and HBRN mice (Fig. 6). This supports previous results with BaP-treated HRN mice (Arlt et al. 2008, 2012).

BaP–DNA adduct formation in the livers of BaP-treated WT, HRN and HBRN mice in vivo (Fig. 6) strongly correlated with the formation of BaP-7,8-dihydrodiol and dG-*N*^2^-BPDE in hepatic microsomal fractions of BaP-pretreated mice (compare Fig. 5a). Collectively, these results support the notion that the Cyb5/Cyb5R systems strongly contribute to BaP–DNA adduct formation in HRN mice.

## Discussion

The in vitro experiments in this study using pooled hepatic microsomal fractions clearly demonstrated that Cyb5 contributes to the bioactivation of BaP: formation of BaP–DNA adducts and BaP metabolites correlated with Cyp1a enzyme activity, and was lessened by the loss of electron donors when NADPH was used as cofactor to examine the POR-dependent pathway. When the NADH-dependent Cyb5R pathway was investigated, there was higher Cyp1a enzyme activity in the microsomal fractions of HRN mice than in those of WT mice. In the microsomal fractions from HBRN mice the activity was significantly lower relative to WT mice, indicating that increased NADH-dependent activity is caused by the increased expression of Cyb5. These results correlate with those from experiments with reconstituted enzyme systems (Stiborova et al. 2014, 2016a) reinforcing the hypothesis that Cyb5 is contributing to the bioactivation of BaP in the livers of HRN mice in the absence of POR.

The present in vivo results, however, contrast with the observations from the in vitro experiments when NADPH was used as cofactor. Whereas the bioactivation of BaP in vitro was reduced with the loss of electron donors and P450 activity, the in vivo results were different. In HRN mice there was a sevenfold increase in hepatic DNA adduct formation compared with WT mice, reflecting findings seen in previous studies (Arlt et al. 2008, 2012). DNA adduct formation in the livers of HBRN mice was significantly lower than in HRN mice, further indicating a role for Cyb5 in the formation of DNA adducts in HRN mice. When NADH was used as cofactor, bioactivation of BaP in vitro (i.e. BPDE–DNA adduct formation) correlated with P450 activity and the formation of BaP-7,8-dihydrodiol (precursor of BPDE) in hepatic microsomal fractions. BPDE–DNA adduct formation in vitro was the highest in hepatic microsomal fraction from BaP-pretreated HRN mice and correlated with the degree of hepatic BPDE–DNA adduct formation in vivo, i.e. highest levels of dG-*N*^2^-BPDE were seen in the livers of BaP-treated HRN mice. These results again support the conclusion that the Cyb5/Cyb5R systems strongly contributes to BPDE–DNA adduct formation in the livers of HRN mice. However, the level of DNA adducts in the livers of HBRN mice was not significantly different from that in the livers of WT mice, which was unexpected.

Both the HRN and HBRN mice display no overt changes in phenotype, although they have steatotic livers as a consequence of non-functioning P450-housekeeping activity involved in cholesterol metabolism (Henderson et al. 2003). There is the potential that the high dose of BaP combined with the steatotic livers could mean that some BaP is retained in the livers and is released and metabolised over a longer period when normally detoxification mechanisms would have allowed any remaining BaP to be excreted. Treatment of mice with doses of 125 mg/kg bw BaP has been shown to be carcinogenic when administered daily for 5 days (Hakura et al. 1998) and is the reason why this dose was used in the present study. A tenfold lower dose of BaP was also found to increase BaP–DNA adduct formation in the livers of HRN mice relative to WT mice (Arlt et al. 2012). Other groups have developed similar models with liver-specific deletion of POR (liver-*Por*-null mouse models) (Wu et al. 2003). It is noteworthy that treatment of liver-*Por*-null mice with 40 mg/kg bw did not result in a significant change in liver retention of BaP relative to WT mice (Wang et al. 2017). Furthermore, in HRN mice the clearance of BaP was not significantly different from that in WT mice (Arlt et al. 2008). BaP clearance in HBRN mice has not been investigated in the present study.

The increased hepatic BaP–DNA adduct formation in the HRN mice indicates that, in accordance with other studies (Shi et al. 2010; Uno et al. 2004, 2006, 2001), P450 activity in hepatocytes in vivo is more important for BaP detoxification than bioactivation. Evidence for this hypothesis came from a study of BaP bioactivation in *Cyp1a1(-*/*-)* mice. Treatment of these mice i.p. with BaP (500 mg/kg bw) resulted in a fourfold increase in hepatic DNA adduct formation relative to WT mice (Uno et al. 2001). Some WT and *Cyp1a1(-*/*-)* in the study were pretreated with 2,3,7,8-tetrachlorodibenzo-*p*-dioxin (TCDD) to assess whether any other dioxin-inducible enzymes were able to contribute to BaP activation. These TCDD-pretreated mice had decreased levels of BaP–DNA adducts and enhanced clearance of BaP from the blood, indicating that the accumulation of BaP–DNA adducts in *Cyp1a1(-*/*-)* mice could be due to the lack of Cyp1a1-mediated detoxification (Uno et al. 2001). Further studies with *Cyp1a1(-*/*-), Cyp1a2(-*/*-)* and *Cyp1b1(-*/*-)* single-knockout, *Cyp1a1*/*1b1(-*/*-)* and *Cyp1a2*/*1b1(-*/*-)* double-knockout mice suggested that Cyp1b1 was responsible for the activation of BaP whilst Cyp1a1 was responsible for detoxification (Nebert et al. 2013). Although these studies provide a rationale for the increased BaP–DNA adduct levels in the livers of HRN relative to WT mice, they do not explain the formation of BaP–DNA adducts in the livers of HRBN mice. These findings may suggest that a P450-independent mechanism contributes to the activation of BaP in HBRN mice. It is noteworthy that DNA adduct formation of the dietary carcinogen 2-amino-1-methyl-6-phenylimidazo[4,5-*b*]pyridine (PhIP) was similar in the livers of reductase conditional null (RCN) and WT mice (Arlt et al. 2011b) whereas RCN mice treated with BaP formed 5.6-fold higher liver DNA adduct levels than WT mice (Arlt et al. 2012). Like HRN mice, RCN mice have a liver-specific deletion of POR and thus show the same phenotype (Arlt et al. 2015a). Although PhIP bioactivation is considered to be catalysed by CYP1A2, the results in the RCN model suggested that PhIP may be activated mainly by a non-P450 pathway in the livers or extra-hepatic P450s of RCN mice (Arlt et al. 2011b).

The existence of a P450-independent BaP activation mechanism has also been suggested by other studies. DNA adduct formation in *AhR(-*/*-)* mice treated i.p. with BaP was similar to WT mice, suggesting an AhR-independent BaP activation mechanism (Kondraganti et al. 2003). When BaP was administered orally to *AhR(-*/*-)* mice, however, DNA adducts levels were significantly higher than in WT mice and a slower rate of clearance was observed (Sagredo et al. 2006), correlating to findings seen in BaP-treated *Cyp1a1(-*/*-)* mice (Uno et al. 2004). Other enzymes have been suggested to be responsible for the bioactivation of BaP as studies have demonstrated a role of prostaglandin H synthase (PTGS/COX) in the oxidation of BaP-7,8-dihydrodiol to BPDE (Marnett 1990; Wiese et al. 2001).

Previous investigations showed that there was no difference in Ptgs1 and Ptgs2 expression between HRN and WT mice when exposed to BaP (Arlt et al. 2008). Hepatic Ptgs2 expression was also compared between *Cyp1a1(-*/*-)* and *Cyp1a1(*+/+*)* mice treated with BaP and no differences were found (Uno et al. 2004). Nevertheless, it may be possible that basal expression of Ptgs/Cox in the livers of HRN and HBRN contributes to the bioactivation of BaP-7,8-dihydrodiol to BPDE, with oxidation of BaP to BaP-7,8-dihydrodiol still needing to be catalysed by a non-P450/non-Ptgs/Cox pathway. Aldo–keto reductases (AKR) have also been implicated in the formation of BaP–DNA adducts, competing with P450 enzymes to activate dihydrodiols. The products of AKR-catalysed reactions are *o*-quinones that produce BaP-7,8-dione–DNA adducts (Huang et al. 2013; Jiang et al. 2005, 2006) distinctly different from those detected in this study. The analysis of BaP-7,8-dione–DNA adducts was beyond the scope of the present study. As indicated above AKRs metabolise dihydrodiols, but not BaP itself, which further questions their involvement in the processes observed in the current study.

Wang and colleagues (Wang et al. 2017) have proposed that, besides Ptgs/Cox and Akr, 5-lipoxygenase contributes to the hepatic bioactivation of BaP in liver-*Por*-null mice. These conclusions were based on in vitro experiments measuring BPDE–DNA adduct formation (i.e. detection of BaP-tetrols by LC–MS/MS analysis after DNA hydrolysis) using S9 fractions isolated from WT and liver-*Por*-null mice and using inhibitors of these enzymes. Although useful, mimicking in vivo phenotypes with inhibition profiles in vitro is not straightforward. First, inhibitors are usually not wholly specific. Second, as seen again in this study using hepatic microsomes, extrapolating in vitro data to in vivo results is often not straightforward. However, it may be appropriate to test some inhibitors of other oxidoreductases (e.g. Ptgs/Cox or Akr) in BaP-treated HBRN mice in vivo and determine the subsequent BPDE–DNA adduct formation in the livers of these animals.

Conversely, our results in BaP-treated HBRN mice may suggest a role for systemic transport of reactive BaP intermediates (e.g. BPDE) to the liver. Besides the liver, other organs (e.g. lung) have been shown to mediate CYP-catalysed bioactivation of BaP (Arlt et al. 2015b). Other studies in mice have demonstrated that blood components (i.e. serum) can facilitate the systemic transport of BPDE (Ginsberg and Atherholt 1989). Thus, the livers of BaP-treated HBRN mice, which are considered largely deficient in CYP-mediated BaP activation, may still receive substantial quantities of DNA-reactive BaP intermediates (i.e. BPDE) or the proximate carcinogen (i.e. BaP-7,8-dihydrodiol) that originate from their CYP-mediated formation in extra-hepatic tissues. It is noteworthy that reactive BaP metabolites have been shown to be transferred from an activator cell to another target cell (Sebti et al. 1982), again illustrating efficient transfer of BPDE despite its reactivity. Other studies have shown that hepatocytes and non-parenchymal liver cells have capacity to catalyse BaP-derived DNA adduct formation (Horton et al. 1985). Thus, it is possible that BaP bioactivation in CYP-expressing non-parenchymal liver cells may contribute to BaP–DNA adduct formation in the livers of HBRN mice. To investigate this, it would require isolation and culture of hepatocytes and non-parenchymal liver cells from HBRN mice and exposure of these cells to BaP for subsequent BaP–DNA adduct analysis.

In summary, this study has shown that both Por and Cyb5 contribute to the bioactivation of BaP in vitro and that Cyb5 also plays an important role in vivo for BaP activation in the HRN mice. The presence of BPDE–DNA adducts in the livers of HBRN mice, however, raises the fundamental question of how BaP is being metabolically activated in the livers of HBRN mice despite the absence of both electron donors. It is clear that HBRN mice possess ample capacity to form hepatic BaP–DNA adducts that arise from BPDE, the same process that occurs in WT mice. The results from this study continue to question the role of P450 enzymes in the bioactivation and detoxification of BaP, as well as to suggest the potential for a P450-independent BaP activation mechanism which will require further investigation. Alternatively our results may also suggest the systemic circulation of DNA-reactive BaP intermediate (i.e. BPDE) which originates from P450-mediated activation in extra-hepatic tissues contributing to the detection of BaP–DNA adducts in the livers of HBRN mice. Likewise, non-parenchymal liver cells may play a role in catalysing P450-mediated bioactivation of BaP in HBRN mice.

## Electronic supplementary material

Below is the link to the electronic supplementary material.

Supplementary material 1 (DOCX 1452 KB)
