# Supplementary material for: Cytochrome b5 impacts on cytochrome P450-mediated metabolism of benzo[a]pyrene and its DNA adduct formation: studies in hepatic cytochrome b5/P450 reductase null (HBRN) mice
Source: Arch Toxicol. 2018 Jan 24;92(4):1625–38. doi: 10.1007/s00204-018-2162-7 (PMC5882632; doi:10.1007/s00204-018-2162-7)
Supplement: Supplementary file 1 — Supplementary material 1 (DOCX 1452 KB) [file 204_2018_2162_MOESM1_ESM.docx]

**Legends to Supplementary Figures:**

**Supplementary Figure 1:**

Enzyme activity in the pooled hepatic fractions of untreated WT, HRN and HBRN mice using either NADPH or NADH as cofactor. (A) POR activity was measured as nmol of cytochrome *c*/mg/min and was detected only in microsomal fractions from WT mice. (B) Cyp1a enzyme activity was determined using the EROD assay with activity being observed as pmol of resorufin/mg protein/min. (C) Cyp1a1 enzyme activity was determined by the oxidation of Sudan I to hydroxylated metabolites with activity being measured as nmol of total C-hydroxylated metabolites/mg protein/min. Values are given as mean ± SD (*n*=3). Statistical analysis was performed by one-way Anova with Tukey’s multiple comparison test (* = compared to WT; # = compared to HRN. * *P* ≤ 0.05 ** *P* ≤ 0.01).

**Supplementary Figure 2:**

(A) Total formation of BaP metabolites during *in vitro* incubations with hepatic microsomal fractions from BaP-pretreated WT HRN and HBRN mice using either NADPH or NADH as cofactor. (B) Total formation of DNA adducts during *in vitro* incubations with hepatic microsomal fractions from BaP-pretreated WT, HRN and HBRN mice using either NADPH or NADH as cofactor. Values are given as mean ± SD (*n*=3). Statistical analysis was performed by one-way Anova with Tukey’s multiple comparison test (* = compared to WT; # = compared to HRN. * *P* ≤ 0.05 ** *P* ≤ 0.01 *** *P* ≤ 0.001 **** *P* ≤ 0.0001).

**Supplementary Figure 3:**

(A) Total formation of BaP metabolites during *in vitro* incubations with hepatic microsomal fractions from untreated WT, HRN and HBRN mice using either NADPH or NADH as cofactor. (B) Total formation of DNA adducts during *in vitro* incubations with hepatic microsomal fractions from untreated WT, HRN and HBRN mice using either NADPH or NADH as cofactor. Values are given as mean ± SD (*n*=3). Statistical analysis was performed by one-way Anova with Tukey’s multiple comparison test (* = compared to WT; * *P* ≤ 0.05 **** *P* ≤ 0.0001).

**Supplementary Figure 4:**

Representative HPLC chromatograms from *in vitro* incubations with hepatic microsomal fractions from BaP-pretreated WT mice with BaP and either NADPH or NADH as cofactor.

**Supplementary Figure 5:**

Structures of BaP metabolites detected from *in vitro* incubations with hepatic microsomal fractions and BaP.

**Supplementary Figure 6:**

Detection of BaP metabolites on HPLC from *in vitro* incubations with BaP and hepatic microsomal fractions from untreated WT, HRN and HBRN mice using either NADPH or NADH as cofactor. Values are given as mean ± SD (*n*=3). Statistical analysis was performed by one-way Anova with Tukey’s multiple comparison test (* = compared to WT; # = compared to HRN. * *P* ≤ 0.05 ** *P* ≤ 0.01 *** *P* ≤ 0.001).

**Supplementary Figure 7:**

Autoradiograms showing adduct profiles by TLC ^32^P-postlabelling analysis from *in vitro* incubations with hepatic microsomal fractions from BaP-pretreated WT, HRN and HBRN mice, DNA and BaP. The origin on the TLC plate, at the bottom left-hand corners, was cut off before exposure. See text for details.

**Supplementary Figure 8:**

Quantitative TLC ^32^P-postlabelling analysis of dG-*N^2^*-BPDE (adduct 1) and adduct 2 from *in vitro* incubations with DNA, BaP and hepatic microsomal fractions from BaP-pretreated or untreated WT, HRN and HBRN mice using either NADPH (A) or NADH (B) as cofactor. Values are given as mean ± SD (n=3). Statistical analysis was performed by one-way Anova with Tukey’s multiple comparison test (* = compared to WT; # = compared to HRN. * *P* ≤ 0.05 *** *P* ≤ 0.001).

**Supplementary Figure 9:**

Autoradiograms showing adduct profiles by TLC ^32^P-postlabelling in organs of WT, HRN and HBRN mice treated i.p with 125 mg/kg bw BaP for 24 h. The origin on the TLC plate, at the bottom left-hand corners, was cut off before exposure. See text for details.

**Supplementary Figure 1**

**Supplementary Figure 2**

**Supplementary Figure 3**

**Supplementary Figure 4**

**Supplementary Figure 5**

**Supplementary Figure 5**

**Supplementary Figure 7**

**Supplementary Figure 8**

**Supplementary Figure 9**
